# Production of β-Lactamase Inhibitors by *Streptomyces* Species

**DOI:** 10.3390/antibiotics7030061

**Published:** 2018-07-17

**Authors:** Daniela de Araújo Viana Marques, Suellen Emilliany Feitosa Machado, Valéria Carvalho Santos Ebinuma, Carolina de Albuquerque Lima Duarte, Attilio Converti, Ana Lúcia Figueiredo Porto

**Affiliations:** 1 Campus Serra Talhada, University of Pernambuco, Avenida Custódio Conrado, 600, AABB, Serra Talhada, Pernambuco 56912-550, Brazil; 2 Department of Antibiotics, Federal University of Pernambuco, Avenida da Engenharia, 2° andar, Cidade Universitária, Recife, Pernambuco 50740-600, Brazil; suellen_feitosa_@hotmail.com; 3 Department of Bioprocesses and Biotechnology, School of Pharmaceutical Sciences, São Paulo State University (UNESP), Rodovia Araraquara-Jaú/Km 01, Araraquara 14800-903, Brazil; valeriac@fcfar.unesp.br; 4 Campus Garanhuns, University of Pernambuco, Rua Capitão Pedro Rodrigues, 105, São José, Garanhuns, Pernambuco 55295-110, Brazil; carolina.albuquerque@upe.br; 5 Department of Civil, Chemical and Environmental Engineering, Chemical Pole, University of Genoa, Via Opera Pia 15, 16145 Genoa, Italy; converti@unige.it; 6 Department of Morphology and Animal Physiology, Federal Rural University of Pernambuco, Av. Dom Manoel de Medeiros, Recife, Pernambuco 52171-900, Brazil; analuporto@yahoo.com.br

**Keywords:** actinobacteria, β-lactamase, resistance, antibiotic, β-lactamase inhibitor

## Abstract

β-Lactamase inhibitors have emerged as an effective alternative to reduce the effects of resistance against β-lactam antibiotics. The *Streptomyces* genus is known for being an exceptional natural source of antimicrobials and β-lactamase inhibitors such as clavulanic acid, which is largely applied in clinical practice. To protect against the increasing prevalence of multidrug-resistant bacterial strains, new antibiotics and β-lactamase inhibitors need to be discovered and developed. This review will cover an update about the main β-lactamase inhibitors producers belonging to the *Streptomyces* genus; advanced methods, such as genetic and metabolic engineering, to enhance inhibitor production compared with wild-type strains; and fermentation and purification processes. Moreover, clinical practice and commercial issues are discussed. The commitment of companies and governments to develop innovative strategies and methods to improve the access to new, efficient, and potentially cost-effective microbial products to combat the antimicrobial resistance is also highlighted.

## 1. Introduction

Infectious diseases, which are caused by bacteria, viruses, parasites, or fungi, persist to be the main cause of mortality worldwide. Despite the success of antibiotics and advances in their production and purification, children and old people are affected by bacterial infections that cause approximately 17 million deaths per year. One of the main reasons for this occurrence is the increasing prevalence of antibiotic-resistant strains [1].

Penicillin was the first antibiotic discovered by Alexander Fleming in 1928, and since then, these compounds have been essential for healthcare [2]. Although the first antibiotic producer discovered was a *Penicillium* strain, many others have this potential, and since 1942, the *Streptomyces* genus has been known for its extraordinary ability to produce secondary metabolites, mainly antibiotics; nearly two-thirds of which occur naturally. This genus is one of the largest genera as it contains over 500 species, described along with a variety of species recognized as Actinomycetes [3,4].

*Streptomyces* are among the most versatile soil microorganisms, given their high metabolite production rate and the large variety of biotransformations. Overall, intracellular mechanisms control the accumulation of metabolites, which depends on process variables, types of nutrients, their concentrations, and operating conditions in submerged culture [5,6]. Therefore, the study and selection of appropriate culture medium composition is essential to ensure high productivity and low costs of the production process [7]. Additionally, many natural antibiotics must be purified after the production process through cost-effective methods that enable one to recover the final product at the highest level of purity and yield [8].

The first *Streptomyces* species used in industrial antibiotic production were *S. griseus* and *S. venezuelae*, which allowed one to obtain streptomycin and chloromycetin, respectively [8]. Since then, a large number of antibiotics (over 55% of available antibiotics) produced by the genus *Streptomyces* were detected between 1945 and 1978 [9].

The first important antibiotic used in clinical practice was penicillin G (benzylpenicillin), a β-lactam compound that attracted the interest of researchers to develop other derivatives. The β-lactam ring present in the structure of this drug, which is typical of this class of antibiotics, acts by linking intimately to the penicillin-binding proteins (transpeptidases), affecting cell wall biosynthesis in both Gram-negative and Gram-positive bacteria [10,11].

Afterwards, as a result of quick replication, recombination, and the high mutation rate of bacteria, resistance to β-lactam antibiotics emerged among the β-lactamase-producing organisms. These enzymes act directly on the β-lactam ring, inactivating the antibiotic. The first β-lactamase was a plasmid-mediated enzyme [12], named TEM from the name (Temoniera) of a patient in which the enzyme-producing *E. coli* strain was isolated. Subsequently, a plasmid with similar biochemical properties was detected and named TEM-2. Faced with this fact, the efforts to discover inhibitors able to inactivate β-lactamases began around 1970. Among them, clavulanic acid, which was obtained by screening of natural products, and possesses a similar β-lactam ring, was found to be a potent inhibitor of staphylococcal penicillinases and other plasmid-encoded penicillinases present in enteric bacteria, including TEM [13].

Nevertheless, antibiotic resistance was not over, and other bacteria began to produce similar enzymes; thus, the main challenge would be discovering novel inhibitors with activity against a broad spectrum of enzymes from multiple classes [10]. Genetic engineering has been an alternative tool to achieve this aim; thanks to a better knowledge of the expression of regulatory proteins in mutant organisms, it suggested that antibiotic production might be influenced by these regulatory events [14].

In accordance with the points explained above, this review provides a summary of the β-lactamase inhibitors produced by *Streptomyces* species. Herein, we emphasize some topics such as antibiotic resistance, β-lactamases inhibitor producers, production and purification processes, use of β-lactamase inhibitors in clinical practice, and commercial aspects.

## 2. Antibiotic Resistance

As previously mentioned, antibiotics produced by *Streptomyces* spp. were detected in 1942 with the discovery of streptomycin, and since then, researchers intensified the search for similar antibiotics within this genus [15].

However, as a mechanism of resistance, bacteria started to produce enzymes with the capacity to hydrolyze β-lactam antibiotics (β-lactamases), hence decreasing their efficiency. Meanwhile, penicillin-resistant strains of *Staphylococcus aureus* and *Streptococcus pneumoniae* emerged, which led to the proposition that the resistance mechanism could be intrinsically associated with the genomes of these bacteria [16,17,18,19,20]. The same phenomenon was observed with Gram-negative bacteria belonging to the *Neisseria* genus [21] and other streptomycin-resistant bacteria [22]. Biofilm formation by microorganisms is also allied with chronic, recurrent human infections and resistance [23]. The spread of resistant strains is linked to the migration of people as well, who dissipate resistant strains among population in remote communities where the use of antibiotics is very restricted [24].

In a bacterial infection, antibiotics act in two stages: in the former, a large part of the bacterial cells are killed, while in the latter, a few of them stay alive and are called persistent bacteria. If the antibiotic is withdrawn, these survivors begin a new multiplication, leading to a slow elimination of infection and recurrent bacterial infections. This phenomenon, which is called persistent sub-minimum inhibitory concentration (MIC), is observed in most bacteria. For instance, tuberculosis, which is caused by *Mycobacterium tuberculosis*, is treated for at least six months, and when the treatment is not continuous, recurrence of infection is commonly observed. Although different from resistant bacteria, the persistent ones are of great concern to the medical field, and the study and knowledge of the mechanisms of this phenomenon are of paramount importance [25].

These sub-MICs suggest a greater selection of resistance, increase bacterial mutation rates, cause phenotypic and genotypic variability, and affect the biofilm formation. In addition, they promote the maintenance of horizontal transmission of resistance genes [26] and can be especially important in multispecies communities, where even small changes in species interaction can have cascading effects [27].

Many Gram-negative bacteria, such as *Haemophilus influenza*; *Klebsiella pneumonia*; *Acinetobacter baumanii*; *Pseudomonas aeruginosa*; *Enterobacter* spp.; *Escherichia coli*; *Serratia* spp.; *Proteus* spp.; *Providencia* spp.; *Helicobacter pylori*; *Salmonella* spp.; *Neisseria gonorrhoeae*; *Shigella* spp.; and some Gram-positive ones such as *Enterococcus faecium*, *S. aureus*, and *S. pneumoniae*, were classified by the World Health Organization (WHO) as “ESKAPE” pathogens, with an extremely important role in the antimicrobial resistance era [28,29].

In addition, another interesting theory was that, unlike antibiotic-producing fungi, *Streptomyces* species defend themselves from antimicrobial attach. These microorganisms possess a self-resistance mechanism to avert the suicide, which made the antimicrobial resistance appear as a natural event preceding the current selective pressure of clinical antibiotic use [30]. The main mechanisms involved in β-lactam antibiotic resistance are inactivation of β-lactamases and modification of penicillin-binding proteins (PBPs). Furthermore, about 90 years after penicillin discovery, β-lactam-based therapies have been largely used against some illness. It is known that the chemical structures of drugs have greatly been modified in the recent past, and this action has become the tactic for most of currently-available antibiotics, which are known as second-, third-, and fourth-generation antibiotics [31,32]. Despite this progress, the indiscriminate use of antibiotics may be responsible for a microbial war in the near future [33].

**Figure 1:**
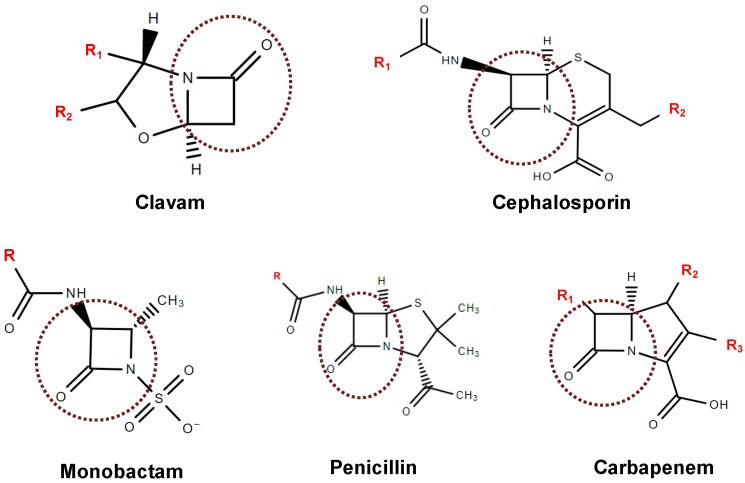
Backbone composition of β-lactam structures with the β-lactam ring highlighted (Structures drawn in ChemSpider).

Beta-Lactam antibiotics were also the first natural antibacterial compounds to be successfully developed and modified by the pharmaceutical industry [34]. This class of compounds has in common a β-lactam four-membered ring associated with different structures (Figure 1). They act by inhibiting bacterial cell wall peptidoglycan biosynthesis, thereby leading to cell lysis and death [35,36]. Specifically, β-lactam antibiotics bind to the acylate active site of PBPs, which is essential for the bacteria cell wall biosynthesis [37].

Beta-Lactam antibiotics have been widely employed for several years to treat infectious diseases because of their high specificity and strong killing efficiency [37]; however, bacterial capacity to develop apparatuses of resistance to β-lactam drugs is one of the big problems of health care [37,38]. Among the mechanisms of antibiotic resistance, we can cite the production of efflux pumps, modification or reduced production of outer membrane porins (in Gram-negative bacteria), alteration of PBPs (the molecular target of β-lactams), and production of β-lactamases [39]. In this review, we will focus on the last resistance mechanism and the mode to overcome this problem by means of biotechnology.

## 3. Producers of β-Lactamases

Because β-lactamases act by breaking the amide bond present in the β-lactam ring of penicillins, they inactivate the drug biological activity, resulting in the inability of antibiotics to kill bacterial cells [40]. The microbial production of these enzymes strongly depends on cell structure. Gram-negative bacteria, with their inner and outer cell membranes separated from the periplasmic space, might be considered more developed microorganisms than the Gram-positive ones, which possess only a rigid cell wall composed of several peptidoglycan layers. As a result, Gram-negative bacteria produce high amounts of β-lactamases compared with the Gram-positive ones. Beta-Lactamase production by the former group can occur by induction (direct interaction of a β-lactam with the microorganism regulatory system) or constitutively, while that by Gram-positive bacteria is not yet well understood [37]. Among the Gram-negative bacteria, the β-lactamase production level can vary greatly; as an example, the active β-lactamase level in some enteric bacteria can exceed 4% of total soluble protein [41].

The enzyme characteristics are quite dependent on the original host. More than 1600 proteins with capacity to hydrolyze β-lactam rings have already been described [42], which, on one hand, demonstrates the diversity of β-lactamase structure [43], but from the other, hinders understanding of their action mechanism [42]. Efforts have been made to classify these enzymes, which were mainly based on their molecular structure (full nucleotide, amino acid sequence, and conserved motifs), functional characteristics (including substrate and inhibition profiles), or variances in structural features [43]. According to Ambler classification, β-lactamases are grouped in classes A, B, C, and D. Classes A, C, and D include enzymes that use serine in their active site to hydrolyze the substrate forming the acyl enzyme, while those belonging to class B are metallo-β-lactamases that require a bivalent ion, usually Zn^2+^, for the hydrolysis [10,44].

On the other hand, Bush, Jacoby, and Medeiros in 1995 [43] classified β-lactamases in four groups based on their functional features: (1) cephalosporinases not fully inhibited by clavulanic acid; (2) penicillinases, cephalosporinases, and broad-spectrum β-lactamases inhibited by active site-directed inhibitors of β-lactamases; and (3) metallo-β-lactamases that are able to hydrolyze the main β-lactam antibiotics, but are scarcely inhibited by typical inhibitors of β-lactamases.

Later, Bush and Jacoby [44] expanded this classification to include subgroups with more recently discovered β-lactamases. As an example, the group 2d comprises the OXA enzymes (oxacillinases) that can hydrolyze cloxacillin and oxacillin [44]. Table 1 summarizes the groups of β-lactamases, while deeper description can be found in the work of Bush and Jacoby [44].

According to the work of Laxminarayan et al. published in 2016, around 214,000 neonatal sepsis deaths worldwide are related to resistant pathogens each year [45]. To overcome the problem of antimicrobial resistance due to β-lactamase production, the pharmaceutical industry has employed two strategies: (i) improvement of β-lactam antibiotics resistant to these enzymes (as an example, the expanded-spectrum cephalosporins and carbapenems); and (ii) the application of β-lactamase inhibitors (BLIs) associated with a β-lactam antibiotic [34]. However, the first strategy has not demonstrated to be so effective because bacteria have developed the capacity to produce enzymes with enhanced features such as extended-spectrum β-lactamases (ESBLs) [40] and carbapenemases [45]. Therefore, application of BLIs may be a valid approach to overcome the problem of antimicrobial resistance.

## 4. Inhibitors of β-Lactamases

**Figure 2:**
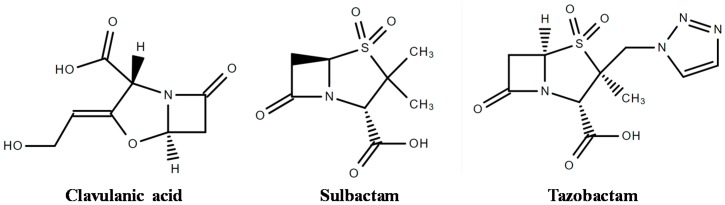
Chemical structure of β-lactamase inhibitors (Structures drawn in ChemSpider).

Although resembling β-lactam antibiotic structure, BLIs are compounds with weak antibacterial activity. Thus, the therapeutic strategy is to co-administer BLIs with penicillins and cephalosporins [36] in two different ways: (i) employing substrates that bind the enzyme reversibly and/or irreversibly, forming unfavorable steric interactions; or (ii) developing mechanism-based or irreversible “suicide inhibitors” as clavulanic acid (CA), sulbactam, and tazobactam (Figure 2) [10,46].

CA is a natural drug isolated in 1976 from *Streptomyces clavuligerus* fermentation broth [41], while sulbactam and tazobactam are BLIs developed by synthetic route in 1980 [40]. CA binds irreversibly with the serine hydroxyl group present in the active site of the enzyme, producing a stable acylated intermediate and inactivating the enzyme. As a result, the antibiotic co-administered with CA performs its main action [47]. Augmentin™ (amoxicillin and potassium clavulanate) [40] and Timentin^TM^ (ticarcillin and potassium clavulanate) [47], both produced by GlaxoSmithKline [10], are examples of combinations available in the market. Drugs combined with CA have proven clinical efficacy against several bacteria (both Gram-negative and Gram-positive) as described by Drawz and Bonomo [10]. CA is associated with other β-lactam antibiotics in its salt form because of its instability under several conditions, such as acidic or alkaline conditions, and in the presence of salts [36].

Sulbactam and tazobactam have inhibitory spectra and mechanisms similar to those of CA [40]. Sulbactam is available on the medicine market in combination with ampicillin (Unasyn^TM^ produced by Pfizer) and cefoperazone (Magnex^TM^ produced by Pfizer) [10]. Sulbactam has advantages compared with other BLIs because it has its own activity against some *Acinetobacter baumannii* strains and does not induce class I (Ampc) chromosomal β-lactamases in Enterobacteriaceae [48]. Moreover, sulbactam combinations have not demonstrated strong selective pressures for ESBL-producing Enterobacteriaceae and vancomycin-resistant enterococci [48].

Tazobactam combined with piperacillin (Zosyn^TM^ produced by Wyeth) has proven clinical efficacy against Gram-positive and Gram-negative pathogens [10]. Tazobactam was also associated with Ceftolozane being an antibacterial agent with potent activity against Gram-negative bacteria, including drug-resistant *P. aeruginosa* and many ESBL-producing Enterobacteriaceae [49]. Another association used to improve the spectrum of antibiotic action is that of piperacillin–tazobactam and vancomycin [50].

Although clavulanic acid, tazobactam, and sulbactam are commercially available BLIs, they exert inhibitory effect against serine-β-lactamases, mainly belonging to classes A, C, and D, which means that they are not inhibitors of metallo-β-lactamases [34]. Regarding metallo-β-lactamases inhibitors, the most important are the following: (i) thiol derivates, such as thiomandelic acids, which bind at the hydrophobic pocket of the enzyme active site and bind to, or interfere with, the bonding network between the hydrolytic water and the Zn^2+^ ions [10]; (ii) dicarboxylate, that is, succinic acid, which binds at the dinuclear metal center using one carboxylate to form a monodentate bridge between both zinc ions, and the second carboxylate to bridge between Zn^2+^ and a conserved Lys residue [51]; (iii) trifluoromethyl ketones and alcohols, the tetrazole portion of whose molecule interacts directly with the active-site Zn^2+^ ions; (iv) carbapenem analogs; (v) tricyclic natural products; and (vi) penicillin derivatives with C-6-mercaptomethyl substituent [10].

Considering that the bacterial resistance is a health care problem, the development of new β-lactamase inhibitors or improvement of already-available BLIs is essential. Meropenem-RPX7009 and Biapenem-RPX7009 by Rempex Pharmaceuticals that are both boronate β-lactamase inhibitors are still in development phase [42]. Although the first citation of boronic acids as serine-β-lactamases inhibitors was already reported many years ago, they have recently been explored as the next generation of pan-β-lactamase inhibitors. Trigonal boron (III) compounds behave as Lewis acids and are prone to react with nucleophiles, resulting in tetrahedral covalent adducts able to resist enzymatic hydrolysis [34]. Another example is Avibactam, which is a diazabyclooctanone approved by the U.S. Food and Drug Administration (FDA) to be used in combination with Ceftazidime (AstraZeneca Pharmaceuticals, Forest–Cerexa, Actavis–Allergan) [42] to treat complicated intra-abdominal infections (cIAI), complicated urinary tract infections (cUTI), and hospital-acquired pneumonia (HAP) [52].

The most part of β-lactamase inhibitors considered in the present review and available in the market is produced through synthetic routes. The exception is CA, which is produced by *Streptomyces* strains by fermentation. However, the development of biotechnology/bioprocesses in the last decades has shown a new perspective to develop new pharmaceutical products or improve already-established processes. Thus, we will present old and new panoramas to overcome the question of antibiotic resistance using biotechnology.

## 5. Producers of β-Lactamases Inhibitors

*Streptomyces* spp. are aerobic, filamentous, Gram-positive bacteria, which resemble fungi and are obtained naturally in different environments. These microorganisms are able to create a chain of spores developing a multicellular complex that, after the sporulation phase, forms hyphae with multinucleated mycelium [53]. These morphological characteristics make *Streptomyces* spp. the most adaptable microorganisms found in the soil, given their broad spectrum of metabolite produced and their biotransformation processes.

The discovery of β-lactamase inhibitors introduced new ways to overcome the problem of antibiotic resistance, through drug research and development [54]. However, as they also contain the same β-lactam ring, they are as susceptible to time-limited application as the β-lactam antibiotics [55]. Resistance to the β-lactam/β-lactamase inhibitor combinations especially of Gram-negative microorganisms is an old and clinically difficult situation [11,56] that has stimulated the use of computation-based design methods to develop new β-lactamase inhibitors [54]. 

As previously mentioned, sulbactam and tazobactam are obtained by synthetic route [54], while clavulanic acid is a secondary metabolite naturally produced by the actinomycete *S. clavuligerus* [57] that belongs to the class of clavams to which it gives its name.

The discovery of clavams, in which an oxygen substitutes a sulfur of penicillins and cephalosporins, occurred during a screening of microorganisms looking for natural products able to inhibit β-lactamases [58,59]. They are structurally related to CA, which, however, is the only one exerting β-lactamase inhibitory activity. Such an activity may be related to the peculiar 3R, 5R stereochemistry of CA, while all the other clavams have a 3S, 5S stereochemistry, although some of them have antibacterial or antifungal characteristics [57,60].

**Figure 3:**
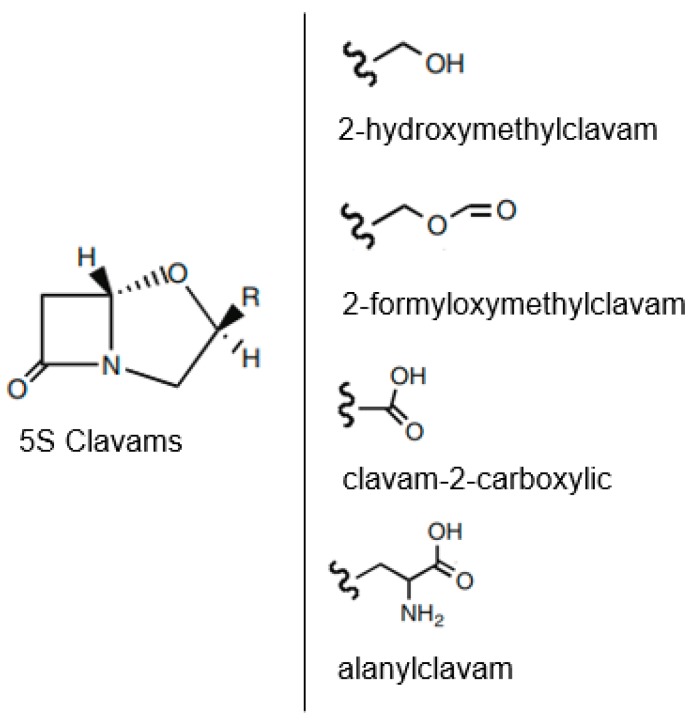
Chemical structure of 5S clavam compounds obtained from *Streptomyces clavuligerus.* (Adapted from Nobary and Jensen [61]).

According to Nobary and Jensen [61], *S. clavuligerus* produces the following 5S clavam compounds in addition to CA: 2-hydroxymethylclavam, 2-formyloxymethylclavam, clavam-2-carboxylic acid, and alanylclavam (Figure 3). Even though not clinically effective, these compounds can be used as precursors to produce CA through a different metabolic route.

CA was identified later in other *Streptomyces* species, namely *Streptomyces jumonjinensis* and *Streptomyces katsurahamanus*, while a great variety of *Streptomyces* species showed the capacity to produce other clavam metabolites with structures similar to CA (clavaminic acid, valclavam, and clavamycins) [62]. More recently, genome-sequencing projects have resulted in deposits of DNA sequences that suggest that CA biosynthetic capability exists in a wider range of strains, including *Streptomyces flavogriseus* ATCC 33331 and *Saccharomonospora viridis* DSM 43017 [61]. The number of species that produce non-CA clavams overpasses that of CA producers, so the ability to biosynthesize and distribute CA is rather limited [62].

Studies investigating biosynthetic ways and genes correlated with the production of CA and other clavams have been performed [63,64,65]. CA biosynthesis involves early and late stages including oxidative reactions catalyzed by 2-oxoglutarate dependent dioxygenases. The “early” steps are largely accepted, but those of the “later” fraction are not understood or are even unknown. Gene clusters for the biosynthesis of CA, 5S clavams, and their paralogs have been identified in *S. clavuligerus* genome sequence. According to Tahlan et al. [66], *ceaS2*, *bls2*, *pah2*, *cas2*, and *oat2* are genes involved in clavaminic acid biosynthesis, thus contributing to both CA and 5S clavams productions, while other genes are only involved in that of CA.

CA obtained by fermentation is then recovered from the medium and purified several times. This production has been the subject of intensive research in recent years because of its clinical and commercial importance [61]. According to Costa and Badino [67] and Ünsaldı et al. [65], CA fermentation processes provide low CA concentrations, which hinders its large-scale chemical synthesis. Moreover, the commercial CA feasibility is restricted because of its elaborate production process [68]. Thus, it is important to get higher production rates of this valuable compound applying more powerful fermentations.

According to Viana-Marques et al. [69], certain nutrients present in the culture medium (such as C, N, P, S sources and salts) and compounds produced in the biosynthetic steps can enhance CA production. Additionally, there are many ways to increase CA production such as optimization of bioreactor operation, conditions of agitation and aeration, and medium composition [70].

After fermentation, the CA-containing fermented broth is clarified by centrifugation and/or filtration [71], and the clarified broth subjected to primary extraction steps involving liquid–liquid extraction followed by adsorption procedures. We will discuss the production and purification process to obtain β-lactamases inhibitors later.

The production of new antibiotics, and investigating and manipulating the biosynthesis routes, represent an important, efficient, and sustainable tool [60], with a chance to discover novel mechanisms of action [72]. To promote the production of new compounds, many strategies have been employed such as cloning and heterologous expression of biosynthetic gene clusters, affecting the regulatory ways, varying culture conditions, and co-culturing two or more organisms together [73].

### 5.1. Natural Microorganisms

The search for bioactive metabolites like novel antibiotics produced by microorganisms for potential use in several industrial applications, mainly in the agricultural and pharmaceutical fields, has become more significant because of the progress of drug/multi-drug resistance in most of pathogenic microbes [74]. Even though several antibiotics used in the clinical practice and agriculture were produced by Streptomycetes, many others were found from natural products produced by this genus and were developed into precious therapeutic agents [62,75].

As mentioned above, *Streptomyces* members are Gram-positive bacteria that grow in different environments with a filamentous form, such as fungi. Soil is the main niche in which they were isolated and identified as prolific producers of effective bioactive compounds other than antibiotics such as antivirals, anticancers, anti-hypertensives, and immunosuppressives [1,74].

*Streptomyces* species were considered as the major producers of bioactive compounds for the biotechnology industry [76]. Genome sequence surveys on various actinomycetes indicate that each bacterium is able to produce about 10-fold more secondary metabolites than the wild type selected by screening analysis before the availability of the genome sequence data. This suggests that actinomycetes are still promising sources of novel bioactive compounds [77].

According to Chater [75], *Streptomyces* are being explored even more intensively in the hope that they will help extensively to the provision of new therapeutic products to face the global threat of antibiotic resistance among pathogenic bacteria, as well as to the supply of other bioactive agents for medical purposes.

More than 23,000 bioactive secondary metabolites obtained from microorganisms have been found, and over 10,000 of them are produced by actinomycetes [78]. In general, the genome sequencing of actinomycetes isolated from soil, or of marine and plant origin, has resulted in the identification of a range of clusters for secondary metabolites, in the quantity of about 20 to 30 clusters per genome. This report reveals unexpected truths in the discovery of new useful agents [79].

Of the 20 (or more) secondary metabolites produced by *S. clavuligerus,* many have clinical importance, like cephamycin C (CephC) and CA, which are synthesized simultaneously through different metabolic routes, rigorously controlling intra- and extracellular factors [80,81].

*S. clavuligerus* is a great model for analyzing the relations involved in the biosynthesis of secondary metabolites because of its productive diversity [82]; for this reason, it has been used in metabolic engineering to obtain new strains able to overproduce CA. Particularly, amplification of biosynthetic genes encoding specific enzymes can conduct to an earlier and more rational method to upgrade strains with higher antibiotic production [62].

### 5.2. Genetically-Modified Microorganisms

Large-scale obtention of antibiotics by microbial fermentation has been the ground of the industry since the development of penicillin in the 1940s. Amounts of these products are nowadays very prominent after years of strong improvement of strategies as such mutagenesis and selection. These strategies, which contribute for strain enhancement, were early adopted for the penicillin strain. For example, Olano et al. reported in 2008 a penicillin production by *Penicillium chrysogenum* higher than 70 g/L, while in 1949, the original strain produced only 60 mg/L, which represents more than a 1000-fold increase [83].

Random mutagenesis and selection techniques are frequently used to obtain the strain most suitable for industrial fermentations, aiming to get high amounts of secondary metabolites [82]. This advance can be realized through over-expression of overall regulators, pathway-specific regulators, or biosynthetic genes. However, genetic engineering attempts to create high-yield strains of a specific product have rarely been successful; thus, enhancing production is still considered a challenge [64].

Furthermore, the deployment of new technologies such as DNA sequencing, transcription profiling, genomics, proteomics, metabolomics, transcriptomics, and metabolite profiling have offered new chances to engineer strains for obtaining high yields of natural products [83].

The improvement of microbial species is also an important way to decrease the production costs of industrial fermentations. Now, the mutation and selection technique is used frequently with success; however, it is very slow and labor-intensive [64].

According to Li and Townsend [82], the creation of a new generation of highly performant strains by this approach may take at least five years. Techniques of molecular genetics have been improved, and the possibility to change existing ways or make non-native pathways has progressed fast (Table 2). Advances in genetics, transcriptional analysis, proteomics, metabolic reconstructions, and metabolic flux analysis offer genetic engineering the chance to enhance the approaches for strain improvement in a targeted way.

Most models of CA overproduction have come from manipulation of genes encoding biosynthetic enzymes or transcriptional regulators [62]. CA is obtained by submerged fermentation and then purified from the fermented broth in different ways; however, the most common protocol is centrifugation for cell separation, followed by liquid–liquid extraction with organic solvents and/or adsorption techniques and, finally, chromatography techniques [85].

## 6. Production of β-Lactamase Inhibitors

### 6.1. Biosynthesis of Clavulanic Acid

Studies carried out by Higgens and Kastner [86] and Brown et al. [87] were the first reports on the production of β-lactamase inhibitors by *S. clavuligerus* ATCC27064 and their recovery from the fermented broth. Although more than 40 years have passed since then, their strategy remains one of the most frequently applied to produce important drugs for medicinal use [88].

**Figure 4:**
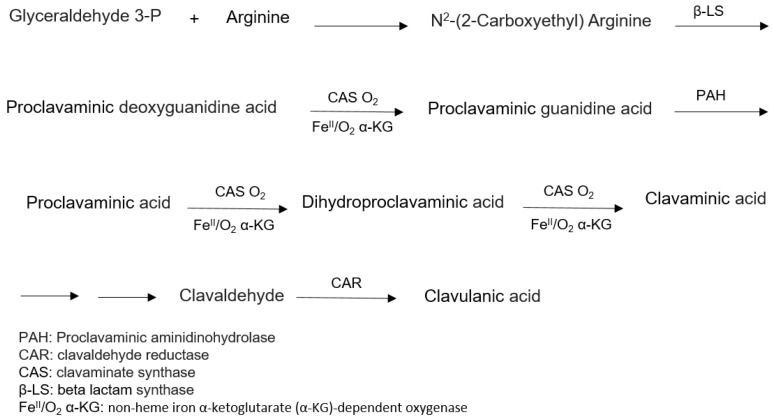
Scheme of clavulanic acid biosynthesis. (Adapted from Oliveira et al. [89,90]).

The biosynthetic pathway to produce CA (Figure 4) has not been fully elucidated, although many intermediates and enzymes have already been isolated. Nonetheless, isotope studies, purification, and characterization of the enzymes involved in the process, together with genetic studies, have contributed to clarify its biosynthetic pathway. Arginine (C5 precursor) and glutaraldehyde-3-phosphate (C3 precursor) were identified as two important precursors for clavulanic acid in *S. clavuligerus* [89,90].

### 6.2. Production Process

After the discovery of β-lactamase inhibitors with antibacterial activity reported by Brown et al. [87], many studies have been carried out in recent decades regarding the production processes of β-lactamases inhibitors [70]. Usually, the industrial production of CA is almost entirely based on *S. clavuligerus* cultivation in complex medium [91]. Because of the clinical importance of this compound, the increase in CA production has been the focus of several studies. Strategies to enhance CA production include optimization of batch or fed-batch operation [92,93], temperature [94,95,96], agitation and aeration [60,93], and medium composition [70,94,95], as well as the selection of new microbial strains [97].

The productivity of microbial metabolites is closely related to the submerged culture process. The selection of the most suitable medium composition is of primary importance to increase the productivity and decrease the cost of any bioprocess [7]. It is well known that extracellular microbial CA production is greatly influenced by medium components, especially carbon and nitrogen sources [70,94], salts composition [70,91,98], and pH [93,94,99]. However, no single medium has been established to optimize CA production by different strains, because each organism requires different conditions for maximum production, which is mainly controlled by intracellular effectors [100].

#### 6.2.1. Carbon Sources

Several carbon sources (glycerol, starch, sucrose, and lipid) have been used to produce CA [70,95,101,102,103]. Glycerol plays an important role in CA production as the C3 precursor of the molecule [104]. The formation of the C3 precursor did in fact appear to be the rate-limiting step of CA synthesis, while excess arginine, which is the C5 precursor, failed to increase CA production [105]. Bellão et al. [80] investigated the effect of carbon source and feeding conditions on the productions of CA and cephamycin C (CephC) by *S. clavuligerus*. In the experimental range studied, glycerol feeding conditions did not influence maximum CephC production (566.5 mg/L), whereas maximum CA concentration (1022 mg/L) was strongly dependent on culture conditions. These results are consistent with those reported by Saudagar and Singhal [93], who obtained higher amounts of clavulanic acid using glycerol in the production medium.

Wang et al. [106] achieved maximum CA production in shake flask batch cultivation at a glycerol concentration of 15.0 g/L, while Teodoro et al. did so in fed-batch culture at 120 g/L [107]. In a fed-batch study carried out in a 10 L bioreactor to determine the influence of glycerol feeding on CA production by *S. clavuligerus*, the highest CA production (1.6 g/L) was obtained using a glycerol concentration of 180 g/L, highlighting a positive effect of glycerol on CA biosynthesis [102]. Many authors have observed that a glycerol concentration above 15 g/L inhibited batch CA biosynthesis [94,100,108]. Viana et al. [94] observed a decrease in CA production by *Streptomyces* DAUFPE 3060 when glycerol concentration was raised from 5 to 10 g/L. The better performance of the fed-batch process compared with the batch one is likely due to its capacity to prevent substrate inhibition and metabolite repression, besides controlling the growth rate and prolonging the stationary phase.

The preferred carbon source for CA production are lipids because of the inability of *S. clavuligerus* to utilize simple carbohydrates such as glucose. Oils are preferred in terms of energy, as they contain approximately 2.4 times the energy of glucose [57]. Several authors have reported that oils may stimulate CA production [93,99,109,110,111,112,113]. Lee and Ho [109] identified palm and palm-kernel oils as the most suitable carbon sources for growth of *S. clavuligerus* and CA production. Large et al. [110] reported maximum CA production (80 mg/L) in a production medium containing unspecified lipids (C16 and C18 unsaturated and saturated fatty acids). Maranesi et al. [111], studying the use of vegetable oil in CA production by *S. clavuligerus* ATCC 27064, found the highest CA concentration (753 mg/L) in a medium containing 30 g/L soybean oil. Saudagar and Singhal [93] obtained similar CA concentrations in media containing palm oil and soybean oil.

Kim et al. [113] investigated the effect of oils on cell growth and CA production during *S. clavuligerus* NRRL 3585 fermentation. Triolein, whose fatty acid is oleic acid only, was the best oil source for CA production, but free fatty acids generated from oil hydrolysis affected both CA production and cell growth. In the same work, the authors screened for *S. clavuligerus* mutants resistant to high oleic acid concentrations and identified a mutant (*S. clavuligerus* OL13) whose oleic acid minimum inhibitory concentration (MIC = 2.1 g/L) was much higher than that of *S. clavuligerus* NRRL 3585 (0.4 g/L). Not only cell growth was improved, but also maximum CA concentration (1,950 mg/L) was approximately twice as high as that of the parent strain.

Efthimiou et al. [112] described an increase in CA production when 47 mg/L olive oil was used instead of 25 mg/L glycerol as the sole carbon source. In a similar study on the effects of different vegetable oil-based media on cell growth and CA production during *S. clavuligerus* ATCC 27064 cultivation, Salem-Berkhit et al. observed that three out of eight tested oils supported CA production [99] and that the olive oil-containing medium ensured a CA concentration twice as high as glycerol-containing medium.

The use of vegetable oils as the sole carbon source can support bacterial growth and enhance CA production, but a careful choice of the oil is essential to prevent affecting the CA yield [112]. These findings can be explained with the residual oil levels in culture medium [114] and the high oxygen requirement for oil metabolism. Residual oil levels may lead to problems associated with the increased medium viscosity and warrant additional downstream processing [93].

Comparing glycerol and sucrose as the sole carbon source, Lee and Ho [109] observed no production of CA in a glycerol-containing medium, but high production in sucrose-containing medium. Similar findings were reported by Ives and Bushell [105], who observed no CA production in glycerol-containing C-limited medium. Another study by Thakur et al. [101] demonstrated that the addition of dextrin or glycerol as the sole carbon source neither improved nor decreased CA production. However, two studies reported a totally different observation, that is, a glycerol-containing basal medium allowed for a maximum CA level (348.5 mg/L) about twice as high as a starch-based medium [80,93].

Additionally, two other studies by Saudagar and Singhal [93] and Chen et al. [100] revealed a biphasic dose response of glycerol, whereby CA production was inhibited at either too high or too low concentrations.

#### 6.2.2. Nitrogen Sources

Soybean derivatives (flour, protein isolate, and meal) have been used as nitrogen sources for CA production [7,67,70,94,95,97,106,107]. To perform a screening of medium constituents for fermentative CA production by *S. clavuligerus*, Rodrigues et al. [70] performed fermentations using soybean protein isolate (SPI) and soybean flour (SF) as the primary nitrogen source, and obtained higher CA concentration (437 mg/L) with the former ingredient. On the other hand, with SF, CA production remained steady for a long time likely because this ingredient induced the release of extracellular proteases by *S. clavuligerus*, which hydrolyzed it during the growth phase, providing a steady supply of essential nutrients to the microorganism [115].

Being a by-product of oil extraction, SF has been recognized as a potentially useful and cost-effective ingredient. It consists of approximately 40% proteins and is rich in other organic and inorganic compounds, thus being a good candidate for a culture medium [116]. According to Chen et al. [102], soybean derivatives, such as soy meal flour and soybean protein hydrolyzates, are excellent components of media for CA production because they contain arginine, the precursor of CA.

Viana et al. [94], in their attempt to investigate the effect of SF concentration on CA production by the new isolate *Streptomyces* DAUFPE 3060, observed maximum CA production (494 mg/L) at the highest level (20 g/L). After optimization by response surface methodology, Marques et al. [95] achieved, with the same strain, a maximum CA concentration as high as 629 mg/L using 40 g/L SF.

Ortiz et al. [7] carried out a study on the influence of the type of soybean derivatives as nitrogen sources on CA production by *S. clavuligerus*. Using two different media, one containing 20 g/L SF and the other 20 g/L SPI, they obtained the highest CA production (698 mg/L) with the former ingredient. On the other hand, Teodoro et al. [107], who investigated the effect of SPI level on CA production, achieved the highest CA yield (380 mg/L) at intermediate SPI concentration (20 g/L corresponding to 2.95 g/L total N).

#### 6.2.3. Amino Acids as Supplements in Basal Medium

Arginine and ornithine exert a concentration-dependent stimulation of CA production, and both amino acids are effectively incorporated into the CA molecule [117,118,119]. The investigation of the role of amino acids as nitrogen sources in CA production began in 1986 [117]. Since then, several studies focusing on the effects of amino acids, mainly arginine and ornithine, have been performed.

The incorporation of arginine into the CA molecule does not establish ornithine as a direct precursor, because the enzyme ornithinecarbamoyl transferase of *S. clavuligerus* exhibits arginase activity, which converts arginine to ornithine [120]. However, Valentine et al. [121] used blocking mutants in the *argF* and *argG* genes, which were unable to convert ornithine into arginine, even though they found a great incorporation of arginine in the CA molecule and a poor incorporation of ornithine. This demonstrates that arginine is the direct precursor of CA and indicates that arginase activity does not produce sufficient ornithine to incorporate into CA.

Townsend and Ho [119] suggested arginine and pyruvate as CA precursors. However, studies have demonstrated that exogenous ornithine, rather than arginine, effectively enhances CA production, provided that there is a sufficient amount of C3 precursor [120]. A 270% increase in CA production was observed intermittently feeding glycerol and ornithine compared with the batch cultivation in shake flasks, and a 150% increase compared with cultures with glycerol and arginine feeding or when only glycerol was fed [122]. Teodoro et al. [102], who investigated the influence of glycerol and ornithine feeding on CA production by *S. clavuligerus* in batch bioreactor, observed an increase in CA productivity, but a small decrease in CA concentration, in the presence of ornithine. Rodrigues et al. [70] observed that glutamate and ornithine negatively affected CA production, while arginine and threonine had no influence.

#### 6.2.4. Salts in Basal Medium

Compounds containing phosphorus, magnesium, and iron are also included in culture media used for CA production [70,106]. Rodrigues et al. [70] published a study on the nutritional requirements of *S. clavuligerus* for CA production where they demonstrated that ferrous sulfate is an essential ingredient of the fermentation medium because the enzymes involved in CA biosynthesis are Fe^2+^-dependent. A medium containing ferrous sulfate allowed for a CA concentration of 437 mg/L, while a formulation without this salt yielded only 41 mg/L.

Phosphate is a crucial growth-limiting nutrient that regulates the synthesis of antibiotics belonging to different groups; therefore, industrial production of antibiotics is carried out at growth-limiting concentrations of inorganic phosphate [57]. Saudagar and Singhal [98] observed that the optimum concentration of KH_2_PO_4_ for CA production (878 mg/L) was 10 mM. According their results, higher CA values are expected at lower temperatures and KH_2_PO_4_ concentrations.

#### 6.2.5. Effect of pH

It has been reported that one of the important characteristics of *S. clavuligerus* is its strong dependence on the extracellular pH for cell growth and CA production [93,94,99]. Viana et al. [94], using a fractional factorial design to investigate the influence of the initial medium pH on CA production by *Streptomyces* DAUFPE 3060, observed the highest CA concentration (494 mg/L) at pH 6.0, while Saudagar and Singhal [93], using a L_25_ orthogonal array, identified pH 7.0 and 7.5 as the optimum pH values for CA production (500 mg/L) and cell growth (140 mg/L nucleic acid), respectively. The marked decrease in the CA yield out of this pH range suggested the occurrence of CA degradation under either acidic or alkaline conditions.

#### 6.2.6. Extractive Fermentation of Clavulanic Acid

CA fermentation processes still present problems such as low CA concentrations [7,92,93,106]; thus, it is essential to search for more effective fermentation methods able to increase the production yield of this valuable compound. Moreover, CA recovery is based on a relatively complex downstream protocol, including successive liquid–liquid extraction steps with organic solvents and a final chromatographic step, which results in low purification yields [68,123]. Therefore, the search for new environmentally friendly (lower amounts of organic solvents) purification strategies is fundamental to achieve higher yields and lower costs. Liquid–liquid extraction in aqueous two-phase systems (ATPS) is an interesting alternative for this purpose [47].

Typical ATPS are the aqueous two-phase polymer systems (ATPPS), formed by two polymers or one polymer and a salt, which separate into two immiscible phases above their critical solubility. Such systems consist of a light phase (top phase), rich in one polymer, and a heavy phase (bottom phase), rich in the second polymer or the salt [124]. Because of the low cost and large difference in hydrophobicity of the two phases, ATPPS, mainly poly(ethylene glycol) (PEG)/salt systems, have been widely used to separate enzymes [125], other proteins [126], and antibiotics [47,68,127].

Some advantages of applying ATPS to whole culture media are the increase in recovery yields, feasibility for continuous operation, reduction of the number of steps, and decrease in the process costs because of joining clarification and partial purification [127]. For most of the ATPS constituted by PEG and salt, the antibiotic is commonly separated into the PEG-rich top phase, whereas the by-products such as amino acids and peptides are discarded into the salt-rich bottom phase [128].

An alternative process has been proposed, which integrates fermentation and extraction with the aim to improve the rate of product formation and production costs. Extractive fermentation or in situ product recovery provides a technological solution to overcome the limitations of product inhibition and low product titer, which are typical drawbacks of biotechnological processes. The concept, as the name suggests, involves integration of an extractive step as the first stage of downstream processing, to remove the product simultaneously to its synthesis. In extractive fermentation using ATPS, cells are considered to be immobilized in one of the phases or at the interface, and the required product to partition into the other phase by proper handling of the system [128].

The advantages of extractive fermentation also include reduction of the toxic effect of product on microbial growth [129] and extended fermentation time [130]. Moreover, continuous product removal during the entire fermentation minimizes the temperature- and pH-dependent degradation of product [131], by reducing its exposure to such damaging conditions. This is particularly beneficial for labile products like CA.

Viana Marques et al. [127] carried out an optimization study according to a 2^2^-central composite design to investigate the influence of four variables, specifically, PEG molar mass, PEG and phosphate concentrations, and agitation intensity, on CA extractive fermentation with a PEG/phosphate ATPS. Whereas CA partitioned towards the PEG-rich top phase, cells positioned at the interface. Moreover, it was found that 25% PEG with molecular weight of 8000 g/mol and phosphate salts at 240 rpm were the best conditions for the extractive fermentation, leading to the best results in terms of CA partition coefficient (*K* = 8.2), yield in the PEG-rich phase (*Y* = 93%), and productivity (*P* = 5.3 mg/L.h).

Panas et al. [132], who attempted the purification of CA produced by *S. clavuligerus* via submerged fermentation using different ATPS, obtained the highest CA recovery yield (64.91%) and purification factor (22.70) with PEG-600/sodium polyacrylate-8000 and PEG-600/cholinium chloride, respectively. These results support the use of these systems as effective techniques to purify CA from fermented broth in a single partitioning step.

## 7. β-Lactamase Inhibitors in Clinical Practice

Immediately after the discovery of β-lactam antibiotics as natural antibacterial compounds, they were effectively modified by the pharmaceutical industry for clinical purposes. However, they even constitute an effective group of antibiotics. With the advance of technologies through rapid bacterial genome sequencing, improvements in protein structure determination, and advanced genetic engineering, many industries started to modify the antibiotic structures leading to the second, third, and fourth generations of these drugs. Nevertheless, the problem of antimicrobial resistance persisted, and combined drug therapies arose [31,32].

Mycobacteria antibiotic resistance is multifactorial, including enzyme inactivation and cell wall impenetrability [133]. Additionally, because this bacteria group developed genetic and epigenetic resistance through selection [132], treatment options for mycobacteria have been restricted and inert for over thirty years [134]. An example is the rapid and global diffusion of genes of strains with multidrug resistance such as NDM-1-producing *K. pneumoniae* [135].

Co-therapy with β-lactam antibiotics and β-lactamase inhibitors, with or without a β-lactam ring, has shown some success (Table 3) [136,137,138]. Oxyimino-cephalosporins or carbapenems are still potentially very reliable and represent a validated strategy to overcome antimicrobial resistance in Gram-negative and Gram-positive bacteria. 

Meanwhile, taking into account that Avibactam (and derivatives) are inhibitors of both class A and class C (and some of class D) serine enzymes, new approaches and new targets are essential to diversify treatment options. The approval of Vabomere (meropenem/vaborbactam) or Avycaz (ceftazidime-avibactam) demonstrates that novel combinations could lead to an amenable successful clinical development.

Boronic acids, which have recently been discovered as a new class of pan-β-lactamase inhibitors, are selective, devoid of toxicity, and able to inhibit both serine- and metallo-β-lactamases. There is a perspective that these inhibitors could be promising and available for clinical use [34].

Phosphonic and phosphinic acids, which contain an inert C–P bond, constitute a group of bioactive small molecules with great pharmaceutical potential. Among the most known examples are fosfomycin (the only FDA-approved antibiotic to treat acute cystitis during pregnancy) and fosmidomycin (a potent antimalarial agent), as well as glyphosate and phosphinothricin (widely used herbicides). However, the use of phosphonic acids as antibiotics/herbicides led to the occurrence of multiple mechanisms of resistance to them [169].

Possible uses of kinase inhibitors in another field, like immune response to bacterial or viral infections, is being investigated in preclinical studies. The receptor tyrosine kinase inhibitor, approved by the FDA, affects *M. tuberculosis* growth through increased lysosomal targeting and suppression of signal transducer and activator of transcription activation [170]. These drugs could also have the ability to inhibit the survival of other microbes and the replication of viruses and, consequently, to decrease the resistance to drugs in patients with infections [171].

## 8. Commercial Use

Despite the emerging need for new antibiotics, there is no advanced research into new antimicrobial drugs. In 2017, only 39 antibiotics were in stages I and III of progress, an amount insufficient to meet the current and planned clinical demand. Further, considering these 39 drugs, only 13 (33%) will be turn into a marketable drug [172]. Because of growing antibiotic resistance and multiresistance, the mortality rate is expected to reach 10 million by 2050, with an estimated economic cost of US $100 trillion [173]. As an example, 250,000 people die every year from the drug-resistant tuberculosis, and only 52% of patients worldwide are successful in treatment. And yet, just two novel antibiotics to treat this disease have reached the market in 70 years.

At first glance, although large companies have some advantages in producing new antibiotics, such as an established research method, sophisticated tools for dosage study, and fast approval by regulatory agencies, they give priority to the development of other drugs for the treatment of other diseases. This occurs because the profit of antibiotic investment is low, representing a market of US $45 billion, losing only to drugs used in cardiovascular and central nervous diseases [174].

A further issue is the antibiotic price when it reaches the market. The competition is great with generic drugs that have a far lower price. Therefore, in some instances, large companies transfer responsibility to small business to develop these drugs; for example, this happens with daptomycin, produced by Cubist and licensed by Lilly [175,176].

The United States and Europe are joining forces to reach the purpose of producing 10 to 15 new antibiotics every decade. This initiative is part of a government program that aims to make US $10–$30 billion available in the market over the next 10 years [177], which would be a real war against drug resistance. Nevertheless, the main obstacle to these strategies is their implementation, as it often implies public acquisition of the rights to distribute the antibiotic that poses a significant risk to companies and major upfront public costs to support this burden.

## 9. Future Perspectives

Nowadays, multidrug resistance is the biggest concern of government agencies and companies that develop antibiotics. Multidrug resistant tuberculosis affects half a million people every year, requires two years of treatment with success in only few cases, and is frequently observed in sites with low human development index. However, many of the novel drugs that are arriving in the market are not directed to infections caused by antibiotic-resistant pathogens. Additionally, according to Pew Charitable Trust studies, only 31% of drugs in advanced stages of clinical trials are effective against an ESKAPE pathogen, and only 33% against the multidrug resistant ones [178].

There are companies and entities around the world that are encouraged to solve the problem of antibiotic resistance. The Global Antibiotic Research and Development Partnership (GARDP), a non-profit organization, is designed to fund research and commercialization of antibiotics. Between 2016–2018, it received funding of more than €5 million for research on new antimicrobial drugs [179].

The isolation of new microorganisms from different environments, such as actinomycetes and more specifically Streptomycetes, able to produce new substances, may considerably accelerate the development of new treatments. Additionally, genetic engineering application and proteomic techniques could generate high performing strains able to produce new bioactive compounds and, consequently, new powerful antibiotics [65].

Besides that, most of the research did not take into account the interactions among bacterial species, which are used to live in communities in the natural environment. In addition, the leakage of antibiotics into natural environments has the potential to radically alter the evolution of resistance along with the microbial dynamics and structure of the communities. Faced with this, the important collaboration of the Amazon Biotechnology Center (CBA) and TB Alliance in the search for new antibiotic producers, and funds to support for the final phase of clinical trials and regulation of using antibiotics, respectively, are a hope [1].

The risk of “super bugs” resistant to relatively all licensed antibiotics may rise in the future; therefore, constant worldwide surveillance for multidrug-resistant bacteria is urgently required. Because of this, strong emphasis on collaboration between companies and governments encourages synergy across the search of new antibiotics and the antibiotic market [1].

## 10. Conclusions

This review focused on a general discussion on the production of β-lactamase inhibitors by the members of the genus *Streptomyces*. Antibiotic resistance has been around for some time and has grown quite a bit as a result of increased mutations in genes encoding enzymes such as β-lactamases, which are responsible for inactivation of β-lactams antibiotics. The classification of these enzymes was reorganized because of the appearance of new genes and, consequently, new enzymes. In view of the fact that antibiotics used in clinical practice at the time were no longer very effective, β-lactamase inhibitors have arisen to circumvent the serious problem constituted by the spread of many extended-spectrum β-lactamases.

Clavulanic acid (CA), the first and most important β-lactamase inhibitor used in clinical practice to date, was obtained naturally by *Streptomyces clavuligerus*, while the other two most known inhibitors, Tazobactam and Sulbactam, are of synthetic origin. The CA industrial production has been extensively studied. Several sources of carbon, nitrogen, vitamins, amino acids, and salts were tested in batch or fed-batch and small or large-scale cultivations. The influence of physicochemical parameters such as pH, temperature, agitation, and aeration on fermentation was also investigated for the purpose of improving CA production and reducing time and costs. The most used purification processes are chromatographic methods, aqueous two-phase extraction using polymers and salts, and extraction systems using solvents. Extractive fermentation is a promising emerging technology that integrates the production and extraction stages, thus increasing productivity and reducing time.

Although *Streptomyces* spp. are known to be excellent producers of antibiotics and β-lactamase inhibitors, rapid and global diffusion of the genes of strains with multidrug resistance is a concern; thus, it is still a considerable challenge to improving production and reducing costs. In this respect, genetic engineering may make it possible to construct combinations of genes capable of coding new, hitherto unknown antibiotics; what we might call hybrid antibiotics.

Recently, bacteria named “ESKAPE” by WHO have emerged, which are extremely important pathogens that spread high levels of resistance across the world. Faced with this, the search for new producers of antibiotics and β-lactamase inhibitors and combined drug therapies are emergency alternatives. For this to occur consistently, companies and government must join forces to develop new low-priced antibiotics to solve the problem of antibiotic resistance.

## Figures and Tables

**Table 1:** Classification of β-lactamases based on Bush and Jacoby [44].

Functional Group According Bush, Jacoby, and Medeiros *		Ambler Class *	Hydrolyzed β-Lactam Antibiotics
1		C	Cephalosporins, benzylpenicillin, cephamycins
1e		C	Ceftazidime and often other oxyimino-β-lactams
2	2a	A	Benzylpenicillin
	2b	A	Benzylpenicillin and cephalosporins
	2be	A	Cefotaxime, ceftazidime, ceftriaxone, cefepime, aztreonam
	2br	A	Resistance to clavulanic acid, sulbactam, and tazobactam
	2ber	A	Oxyimino-β-lactams combined with resistance to clavulanic acid, sulbactam, and tazobactam
	2c	A	Carbenicillin
	2ce	A	Carbenicillin, cefepime, and cefpirome
	2d	D	Cloxacillin or oxacillin
	2de	D	Cloxacillin or oxacillin, and oxyimino-β-lactams
	2df	D	Cloxacillin or oxacillin and carbapenems
	2e	A	Cephalosporins inhibited by clavulanic acid
	2f	A	Carbapenems, oxyimino-β-lactams, cephamycins
3		B	Carbapenems
4		Unknown	Carbapenems

**Table 2:** Genetic methods applied to improve the production of antibiotics and β–lactamase inhibitors (Adapted from Adrio and Demain [84]).

Genetic Method	Secondary Metabolite
Protoplast fusion	Penicillin G, cephalosporin C, cephamycin C, clavulanic acid, indolizomycin, rifamycins
Metabolic engineering	Antibiotics (penicillin G, cephalosporin C, cephamycin C, clavulanic acid, semisynthetic cephalosporins)
Transposition	Daptomycin, tylosin
Combinatorial biosynthesis	Erythromycins, tetracenomycins, tylosin, spiramycins
Genome mining	Echinosporamicin-type antibiotics, antifungal compounds (ECO-02301), and so on

**Table 3:** Combinations of β-lactamase inhibitors and β-lactam antibiotics of clinical use (Adapted from Docquier and Mangani et al. [34] and Bush [41]).

β–Lactamase Inhibitor	β–Lactam Antibiotic	Development Status	References
Clavulanic acid	Amoxicillin	Approved by FDA, EMA	[139]
Sulbactam	Ampicillin	Approved by FDA, EMA	[140]
	ETX2514	Phase 1 trials completed in 2017	[34]
Tazobactam	Piperacillin	Approved by FDA, EMA	[141]
	Ceftolozane	Approved in 2014 by FDA and in 2015 by EMA	[34,142,143,144,145,146]
	Cefepime	Used in Asia	[147]
Avibactam	Ceftazidime	Approved in 2015 by FDA and in 2016 by EMA	[34,148,149,150,151,152,153,154,155,156,157,158,159]
	Aztreonam	Phase 2 in progress	[149,153,160,161]
	Ceftaroline	Phase 2 in progress	[149,159,162,163]
Relebactam (MK7655)	Imipenem (+ cilastatin)	Phase 3, cUTI (in progress) Phase 2 cIAI (completed)	[164,165]
Vaborbactam (RPX7009)	Meropenem	Approved in 2017 by FDA	[34,166]
AAI101	Cefepime	Phase 2 initiated in 2017	[34,167,168]
RG6080 (OP0595, FPI-1459)	Unknown	Phase 1 complete	[165]

FDA, U.S. Food and Drug Administration; EMA, European Medicines Agency; cUTI, complicated urinary tract infection; cIAI, complicated intra-abdominal infection.
